# Perception of Verticality and Vestibular Disorders of Balance and Falls

**DOI:** 10.3389/fneur.2019.00172

**Published:** 2019-04-03

**Authors:** Marianne Dieterich, Thomas Brandt

**Affiliations:** ^1^German Center for Vertigo and Balance Disorders, Ludwig-Maximilians University, Munich, Germany; ^2^Department of Neurology, Ludwig-Maximilians University, Munich, Germany; ^3^Munich Cluster for Systems Neurology, Munich, Germany; ^4^Clinical Neuroscience, Ludwig-Maximilians University, Munich, Germany

**Keywords:** vertical orientation, subjective visual vertical, subjective postural vertical, vestibular system, graviception, hemispatial neglect, pusher syndrome

## Abstract

**Objective:** To review current knowledge of the perception of verticality, its normal function and disorders. This is based on an integrative graviceptive input from the vertical semicircular canals and the otolith organs.

**Methods:** The special focus is on human psychophysics, neurophysiological and imaging data on the adjustments of subjective visual vertical (SVV) and the subjective postural vertical. Furthermore, examples of mathematical modeling of specific vestibular cell functions for orientation in space in rodents and in patients are briefly presented.

**Results:** Pathological tilts of the SVV in the roll plane are most sensitive and frequent clinical vestibular signs of unilateral lesions extending from the labyrinths via the brainstem and thalamus to the parieto-insular vestibular cortex. Due to crossings of ascending graviceptive fibers, peripheral vestibular and pontomedullary lesions cause ipsilateral tilts of the SVV; ponto-mesencephalic lesions cause contralateral tilts. In contrast, SVV tilts, which are measured in unilateral vestibular lesions at thalamic and cortical levels, have two different characteristic features: (i) they may be ipsi- or contralateral, and (ii) they are smaller than those found in lower brainstem or peripheral lesions. Motor signs such as head tilt and body lateropulsion, components of ocular tilt reaction, are typical for vestibular lesions of the peripheral vestibular organ and the pontomedullary brainstem (vestibular nucleus). They are less frequent in midbrain lesions (interstitial nucleus of Cajal) and rare in cortical lesions. Isolated body lateropulsion is chiefly found in caudal lateral medullary brainstem lesions. Vestibular function in the roll plane and its disorders can be mathematically modeled by an attractor model of angular head velocity cell and head direction cell function. Disorders manifesting with misperception of the body vertical are the pusher syndrome, the progressive supranuclear palsy, or the normal pressure hydrocephalus; they may affect roll and/or pitch plane.

**Conclusion:** Clinical determinations of the SVV are easy and reliable. They indicate acute unilateral vestibular dysfunctions, the causative lesion of which extends from labyrinth to cortex. They allow precise topographical diagnosis of side and level in unilateral brainstem or peripheral vestibular disorders. SVV tilts may coincide with or differ from the perception of body vertical, e.g., in isolated body lateropulsion.

## Introduction

The perception of verticality in the roll and pitch planes is based on an integrative graviceptive input from the vertical semicircular canals and otolith organs. This input is mediated by a bilateral central circuitry connecting the vestibular nuclei with integration centers for vertical and torsional eye-head coordination located in the rostral midbrain tegmentum (interstitial nucleus of Cajal, INC; rostral interstitial nucleus of the medial longitudinal fascicle, riMLF) and the thalamus (in particular, the paramedian and dorsolateral subnuclei). The vestibular input has to be integrated with visual and somatosensory information about vertical orientation of the three-dimensional space relative to the earth-centered gravitational force. Especially, the visual and the vestibular systems provide us with information about vertical orientation. Its coordinates have to be matched by convergence to create the actual global percept of up and down, right and left, and fore and aft. This percept may apply to either the egocentric orientation of surrounding targets or to the allocentric orientation of body position within the environment. The sensory modalities involved cannot perceive different verticals at the same time independently—a visual and a vestibular one. This multisensory input establishes an internal model of space and verticality, which is updated via bottom-up and top-down processes (1, 2). Other models use Bayesian spatial-perception (3–5) and an inverse probabilistic approach based on an optimal observer theory (6).

With respect to orientation in space, vestibular input from the otolith organs in stationary subjects enables a two-dimensional (egocentric) spatial orientation, input from the semicircular canals and otolith organs in mobile subjects contributes to a three-dimensional (allocentric) spatial orientation. The novelty of such a concept is that two reference frames—”egocentric and allocentric”—are attributed to two operational modes—“static and dynamic” (7). An explanation involving a strictly dichotomous separation, however, is too simple, since both reference frames and modes of operation have to be integrated according to the particular task in natural environments. Thus, tests of vestibular function (in virtual or real environments) involve a static, two-dimensional and a dynamic, three-dimensional mode of action, respectively (7).

In the current clinical review we focus on psychophysical adjustments of the subjective visual vertical (SVV) and the subjective postural vertical (SPV) for balance control in a three-dimensional space. Depending on the method employed, different sensory systems come into play when the subjective vertical is assessed. The clinical examination of body orientation in space is performed in heterogeneous ways of measuring the body vertical (e.g., a moving chair on a platform or the three-axes space curl), the haptic vertical (metal rod), and the visual vertical (with several devices, e.g., use of spectacles or adjustments of visual lines at some distance in front of the body). These different approaches aim to quantify the input of different senses such as the somatosensory sense from the trunk and the lower limbs (body postural vertical, haptic vertical), the vestibular sense (subjective visual vertical without visual cues for orientation), and the visual sense. However, no known measure can be solely attributed to only one sensory system. The brain seems to use Bayesian inference to integrate noisy multisensory signals to reduce perceptual uncertainty by weighting the signals in proportion to their reliability (6, 8).

Other modalities can in part substitute for the deficit of patients with disorders of a particular sensory function. For example, patients with spinal cord injuries (lacking somatosensory input from the lower body) perceive verticality without any significant directional bias in orientation, both in haptic and postural tests, but they are more uncertain than control subjects (9). Similar findings were reported for patients with peripheral vestibular disorders (10, 11). Thus, humans create and update internal models of verticality on the basis of convergence and integration of vestibular, somatosensory, and visual graviceptive cues. The posterolateral thalamus seems to play a crucial role in this process of integration of vestibular and somatosensory input (12). It is increasingly acknowledged that the role of the thalamo-cortical system with its widespread connectivities is much broader. The thalamus has even been termed a multisensory and cognitive integrative hub that encompasses spatial orientation and motion perception (13, 14).

## Methods of Verticality Perception

### Subjective Postural Vertical (SPV)

To assess the *postural vertical* the subject sits on a tilting device in darkness and adjusts himself in a vertical position. For example, the seat of the blindfolded participants is tilted to the left or right relative to gravity and they are then asked to adjust the tilt of the motion base until they feel upright (10, 15–18). Another method in which the subject stands is the space curl, a three-axis system similar to a gyroscope (19). This device was also used for rehabilitation of verticality perception [e.g., in pusher syndrome (20)].

To assess the *subjective haptic vertical*, a subject sitting in the dark adjusts a rotatable bar by his tactile sense until it is vertical (21).

### Subjective Visual Vertical (SVV)

Measurement of the perceived visual vertical discloses acute unilateral vestibular dysfunction when the device used provides no cues to visual spatial orientation as in darkness or with a random dot pattern background (22–24). A systematic review of visual vertical assessment methods showed a great heterogeneity of the parameters, settings, and procedures. Only a few are suitable for standardization so as to limit errors and improve interpretation of the results (25). This review assessed data of 61 studies (1,982 patients) on SVV measurement procedures for hemispheric (*n* = 43), brainstem (*n* = 18) or cerebellar (*n* = 8) strokes (25). SVV assessment procedures varied in paradigm, type of stimulus, patient posture, number of trials and results. Therefore, the authors recommended that the SVV be assessed in darkness and in an even number of trials (6 to 10) with the body in an upright position. Then, normal SVV orientation (mean of SVV adjustments) can be considered to range from −2.5 to 2.5° and is reliable for clinical use and research studies. This corresponds to the normal ranges for measurements with a hemispheric dome (22).

In the *hemispheric dome* method (22), patients sit in front of a device which covers the entire visual field and its inner surface presenting a random pattern of colored dots that provides no cues to true vertical orientation. Participants are asked to move a linear target located at random offset positions into a vertical position in the center of the dome.

In the *bucket test* (26, 27) the subjects evaluate the vertical orientation by properly aligning a straight line visible on the inner bottom of the bucket which the examiner rotates at random. On the outer bottom surface of the bucket an angular protractor provides the examiner to readout the tilt angle.

In the computerized *Visual-Spatial Perception Program* (28) the SVV procedure expects the subject to vertically orient a tilted white line on a dark background.

### Differentiation of Vestibular and Peripheral Ocular Motor Disorders

Some caution is required in choosing the appropriate device for SVV measurements. In certain studies the visual vertical was measured by using glasses similar to a Maddox double rod *directly in front of the eyes*. The problem of this technique, adopted from ophthalmologic labs, is that it determines the subjective perception of the cyclorotation of one eye, for example, in extraocular eye muscle palsies, rather than the perceived vertical of the visual environment. Measurements with the monocularly and binocularly determined SVV using a device in front of the body, the subjective perception of ocular torsion, or the objective determination of ocular torsion with fundus photographs yielded different results [for review see: (29)]. For example, the monocular SVV of the right eye of a patient with an acute right third nerve palsy showed a pathological tilt of +19°, whereas the SVV of the left eye and the binocular SVV were both normal (−1.6°, −2.0°). The Maddox double rod gave a right excyclotropia of 4°-5°, and the fundus photographs, an excyclotropia of 8° right (normal) and 7° left (normal). This example clearly demonstrates that a valid way of distinguishing between central vestibular lesions and extraocular eye muscle paresis (third or fourth nerve palsy) is the dissociated occurrence of SVV tilts and ocular torsion in both the non-paretic and the paretic eye. The SVV tilts of patients with eye muscle pareses occur only during monocular testing; tilts are normal during binocular testing (29, 30). Thus, monocular vs. binocular measures of SVV tilt allow us to differentiate vestibular from peripheral ocular motor disorders.

### Disorders of the Postural Vertical

Misperception of the postural body vertical is critical for hemispheric and thalamic disorders such as the pusher syndrome (17, 18, 31) and the idiopathic normal pressure hydrocephalus (NPH)(32) as well as brainstem disorders such as the progressive supranuclear palsy (PSP) syndrome (33, 34) and the dorsolateral medullary Wallenberg syndrome (35). A misperception in the frontal roll plane is typical for the *pusher syndrome* which also includes lateral falls (17, 18). Such patients have a severe misperception of their body's orientation and experience it as “upright” although it is tilted. The thus afflicted patients actively push the body away with the unparalysed arm or leg to the contralateral side. Patients with pusher syndrome cannot correctly indicate their own body's upright. However, they appear to have no difficulty to determine the vertical orientation of the visual surrounding (17, 18).

The perception of upright body orientation in the pusher syndrome was also investigated while the patient was standing in the space curl device. The study revealed that these patients adjusted their body with an ipsilateral lateral tilt in the roll and also in the pitch plane, an adjustment that decreased with decreasing severity of the condition (36). Their uncertainty in the perception of verticality in both roll and pitch planes indicates a global misperception of verticality.

Causative lesion sites may include the thalamus and—perhaps more likely—the posterior insula (31, 37). Components of the multisensory cortical vestibular network are located at these sites. The right hemispheric dominance in this network corresponds to the significantly higher frequency of the pushing syndrome in strokes of the right hemisphere (38), an observation that explains the clinical experience of physical therapists, that recovery from pushing behavior takes longer after right- compared to left-hemispheric strokes (39).

A misperception of body verticality in the sagittal pitch plane is typical for patients with idiopathic *normal pressure hydrocephalus (NPH)*. Such misperception was considered a potential diagnostic tool (and a therapeutic predictor) for these patients before and after cerebral spinal fluid drainage. A correlation was found between the backward tilt of the subjective body vertical and a ventricular enlargement of the frontal horns neighboring the thalamic nuclei. Thus, such a disturbance in the pitch plane might indicate a bilateral vestibular dysfunction of the thalamus; it promises to increase diagnostic accuracy of suspected NPH (32).

Postural instability in the pitch plane has also been documented in neurodegenerative *progressive supranuclear palsy* (PSP), especially the occurrence of backward falls in early stages of the disease (34). In addition to early postural instability with falls, PSP is defined by supranuclear vertical gaze palsy, bilateral akinesia and muscle rigidity as well as frontal and subcortical dementia with pseudobulbar palsy (33, 40). Postural instability leads to gait abnormalities like freezing that can be quantitatively characterized (41, 42). Patients who self-monitored the frequency of falls, underwent a standardized clinical investigation, posturographic analysis of balance during experimentally modified sensory input, and a [18F]FDG-PET. Further, they performed an fMRI paradigm that involved mental imagery of upright stance. Compared to age-matched controls sway path values were higher and the frequency of falls was associated with decreased cerebral regional glucose metabolism (rCGM) of the thalamus, but increased rCGM of the precentral gyrus. In the fMRI mental imagery of stance induced a decreased activation of the mesencephalic brainstem tegmentum and the thalamus in those patients with postural imbalance causing falls. Thalamic dysfunction of postural control was most evident when balance was assessed during modification of the actual sensory input (41). The results support the view that reduced thalamic activation by ascending brainstem projections causes postural instability in PSP (34). Thus, gait impairment in PSP indicates dysfunction of the indirect, prefrontal-subthalamic–pedunculo-pontine loop for control of balance and locomotion. The stereotyped, direct locomotor loop connecting the primary motor cortex and the spinal cord (with rhythmic cerebellar drive) revealed an increased activity in PET during walking (42). This can be explained as an attempted compensation or a contribution to the stereotyped gait pattern in PSP.

In quantitative gait analyses patients with PSP are more sensitive to perturbations performing dual tasks than patients with NPH. Cognitive dual-tasks caused a more pronounced reduction of gait velocity in PSP. Motor dual-tasks resulted a dissociation in locomotion performance in both disorders: it worsened considerably in PSP patients, but tended to improve in NPH patients (43).

### Isolated Body Lateropulsion

The phenomenon of *axial body lateropulsion* occurs when the body is pulled toward the lesion side and there is a tendency to fall down. It is a well-recognized transient feature of a lateral medullary syndrome (44–46) and axial body lateropulsion may occur in some patients even without vestibular and cerebellar dysfunctions (isolated body lateropulsion). They suffer from a caudal medullary lesion of the spinocerebellar tract, the descending lateral vestibulospinal tract, the ascending vestibulo-thalamic and dentatorubro-thalamic pathways, or the thalamocortical fascicle (44, 45, 47, 48). The isolated symptomatology of lateropulsion can be attributed to lesions below the network of the vestibulo-ocular reflex (VOR), which links the extraocular eye muscles and contributes to the perception of gravitational vertical. In very rare cases cortical strokes of the parietal lobe can also cause isolated or predominant body lateropulsion like those of the posterior cingulate and/or precuneus (49).

More often patients with acute lesions of the medullary brainstem, especially dorsolateral medullary infarctions (i.e., Wallenberg syndrome including the vestibular nuclei) present with lateropulsion and additional vestibular signs such as a deviation of the SVV, skew deviation of the eyes, and ocular torsion, all of which are directed to the ipsilateral side (38, 50). It is striking that these patients in the postacute phase do not experience subjective vertigo, despite their strong tendency to fall sidewards (35). This can be explained by postural regulation, which aims to adjust the body to the tilted vertical. Lateropulsion can be interpreted as a postural compensation of an erroneously perceived body tilt contralateral to the side of the lesion. Despite the thus elicited postural imbalance and the conflicting true vertical, the posture is continuously pushed toward what the central nervous system wrongly computes as being vertical (50). The extent of the damage of vestibular structures can certainly vary; in single cases a combination of isolated axial lateropulsion with only ipsilateral SVV tilts was reported in small caudal medullary lesions (46).

### Disorders of the Visual Vertical

**Figure 1:**
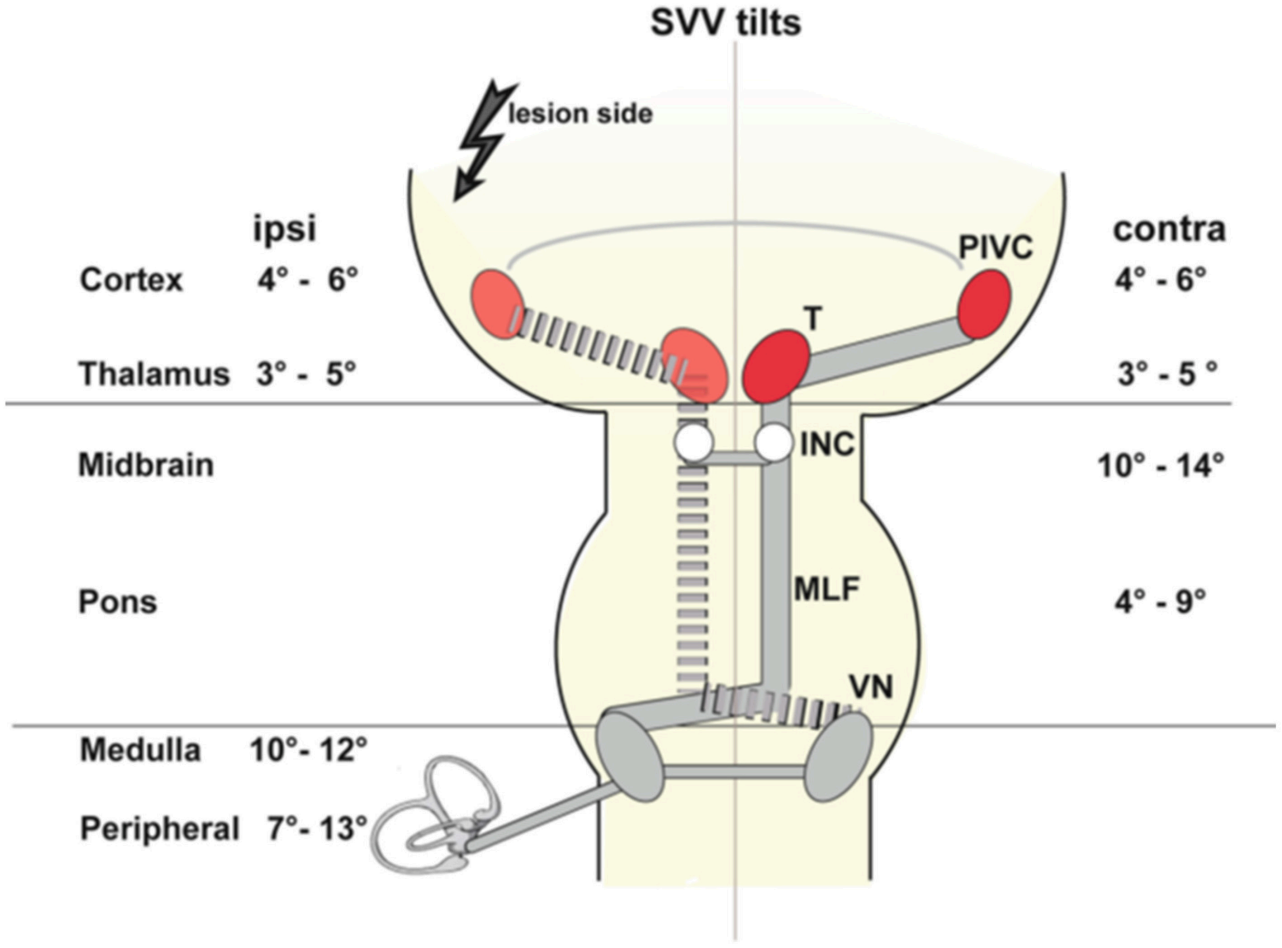
Schematic graviceptive pathways together with the amount of SVV tilt (in deg) for ipsilateral (ipsi) and contralateral (contra) lesions depending on the level of acute unilateral vestibular damage. The range of the mean values was calculated from a total of 15 published studies (see Table 1 for reference numbers). The four major messages are as follows: (i) In peripheral and pontomedullary brainstem lesions SVV tilts are ipsilateral. (ii) In pontomesencephalic vestibular pathway lesions up to the INC, SVV tilts are contralateral. (iii) In vestibular thalamic and cortical lesions, SVV tilts may be either ipsilateral or contralateral with an intraindividual consistency and an equal distribution interindividually. (iv) The amount of SVV tilt is maximal in complete peripheral lesions (mean up to 13 deg) and in brainstem lesions (mean up to 12–14 deg), and less in lesions of the vestibular thalamus and cortex (mean up to 5–6 deg). INC, interstitial nucleus of Cajal; MLF, medial longitudinal fascicle; VN, vestibuar nucleus [From Glasauer et al. (2)].

Tilts of SVV are the most frequent sign of an *acute tone imbalance* of the bilateral vestibular system in the roll plane. They occur with acute unilateral lesions of the graviceptive pathways that originate from the otolith organs and the vertical semicircular canals and travel via the vestibular nuclei and the vestibular subnuclei of the thalamus to the parieto-insular vestibular cortex, PIVC (Figure 1). Adjustments of SVV are ipsilateral in peripheral and caudal ponto-medullary brainstem lesions but contralateral in ponto-mesencephalic lesions (2, 22, 23, 25, 51–53). Lesion sites along the brainstem pathways were confirmed more recently by voxel-wise lesion-behavior mapping techniques in MRI (52, 53). In contrast, unilateral lesions of vestibular thalamus or cortex areas manifest with smaller tilts of SVV, and—importantly—can be either ipsilateral or contralateral (2, 25, 54–56) (Figure 1, Table 1).

**Table 1:** SVV tilts in acute unilateral vestibular lesions at different lesion sites from labyrinth to cortex (i, ipsilateral tilt; c, contralateral tilt).

	**TSL**	**No. of**	**Amount of SVV tilt [deg]**			**References**
	**[days]**	**Patients**	**Mean/median**		**(Range)**	
			**ipsi**	**contra**		
Cortex	1–7	54			(i −8.7; c −7.5)	(37)
	2–12	52	i 4	c 3.4–6.2	(2.7–15)	(55)
	4–10	82	i 5.4	c 5.3		^#^
Thalamus	1–9	37	i 3.4	c 5.1		(57)
	1–7	17	i 3	c 4		(54)
Midbrain	1–9	14		c 13.5		(54)
	1–15	28			(6–29)	(22)
Ponto-mes.^*^	−14	14	i 4.1		(2.7–6.6)	(58)^*^
Pons	1–15	47	i 9.3		(5–15)	(22)
Medulla	1–5	36	i 11		(5–22)	(35)
	1–15		i 12.4			(22)
	1–10	50	i 9.8		(−28)	(59)
	1–2	43	i 7.9			(60)
Brainstem	i 1–19	82	i 7.0	c 4.2		(53)
in total	c 2–8					
	3–9	79	i 4.5	c 4.2	(2.3–9.6)	(52)
	1–10	111	i/c 8.1		(2.7–26)	(22)
**LABYRINTH/NERVE**						
Neuritis	1–11	50	i 7		(−25)	(59)
Neuritis	1–2	40	i 7.3			(60)
Neuritis	3–4	5	i 12.2		(5.5–33.3)	(61)
Neuritis	1–14	20	i 6.8		(0.2–33.0)	(62)
Neurectomy	1–10	13	i		(10–30)	(63)
Neurectomy	1–7	5	i 8.5		(7–10)	(10)
Neurectomy	4–10	13	i 11.9		(6.6–22)	(64)
Neurectomy	1–14	15	i 12.4		(4.8–21.4)	(62)
Labyrinthect.	1–7	6	i		(4–21)	(65)
Zoster	1–7	4	i 10.4		(3.2–17.2)	(62)

*TSL time since lesion onset; ipsi = i = ipsilateral tilt; contra = c = contralateral tilt*.

* *tilts in ponto-mesencephalic lesions are typically due to an affection of the medial longitudinal fascicle (MLF) which crosses midline above the vestibular nuclei and therefore show contralateral directions of tilts, skew deviation and ocular torsion. One exception from that rule has been described for rare anteromedian pontomesencephalic lesions close to and within the medial lemniscus which manifested with isolated ipsilateral SVV tilts [without skew deviation and ocular torsion; Zwergal et al. (58)]*.

#, *Baier et al., unpublished*.

TSL, time since lesion onset; ipsi = i, ipsilateral tilt; contra = c, contralateral tilt.

Cerebellar lesions may also cause vestibular dysfunction in the roll plane. Acute unilateral lesions of the vestibulo-cerebellar loop induce either ipsilateral or contralateral SVV tilts depending on the cerebellar lesion site (66). However, the amount of tilt is larger than in thalamo-cortical lesions and more in parallel to those of medullary brainstem lesions and have an identical time course (67). MRI lesion mapping in patients showing contralateral SVV tilts (in some patients a complete OTR) disclosed the dentate nucleus as the causative structure. In contrast, ipsilateral tilts indicated lesions in the biventer lobule, the middle cerebellar peduncle, the tonsil and the inferior semilunar lobule, sparing the dentate nucleus (66).

The *spontaneous course* of SVV tilts indicates that they are due to an *acute* vestibular dysfunction. They most often decrease and normalize over time within a few weeks. In patients with a unilateral lesion of the dorsolateral medulla affecting the vestibular nucleus the deviations recovered within about 4 weeks (22, 35, 59). A comparable time course of SVV tilts was seen in patients with an acute vestibular neuritis, now termed acute vestibular syndrome (59). Patients with an acute unilateral cerebellar infarction also had a spontaneous recovery within 2–4 weeks (67). The MRI of some patients who had a pathological deviation of the perceived vertical lasting several months or years revealed damage to the cerebellar structures necessary for compensation and recalibration (68).

### SVV Tilts and Associated Vestibular Motor Signs

**Figure 2:**
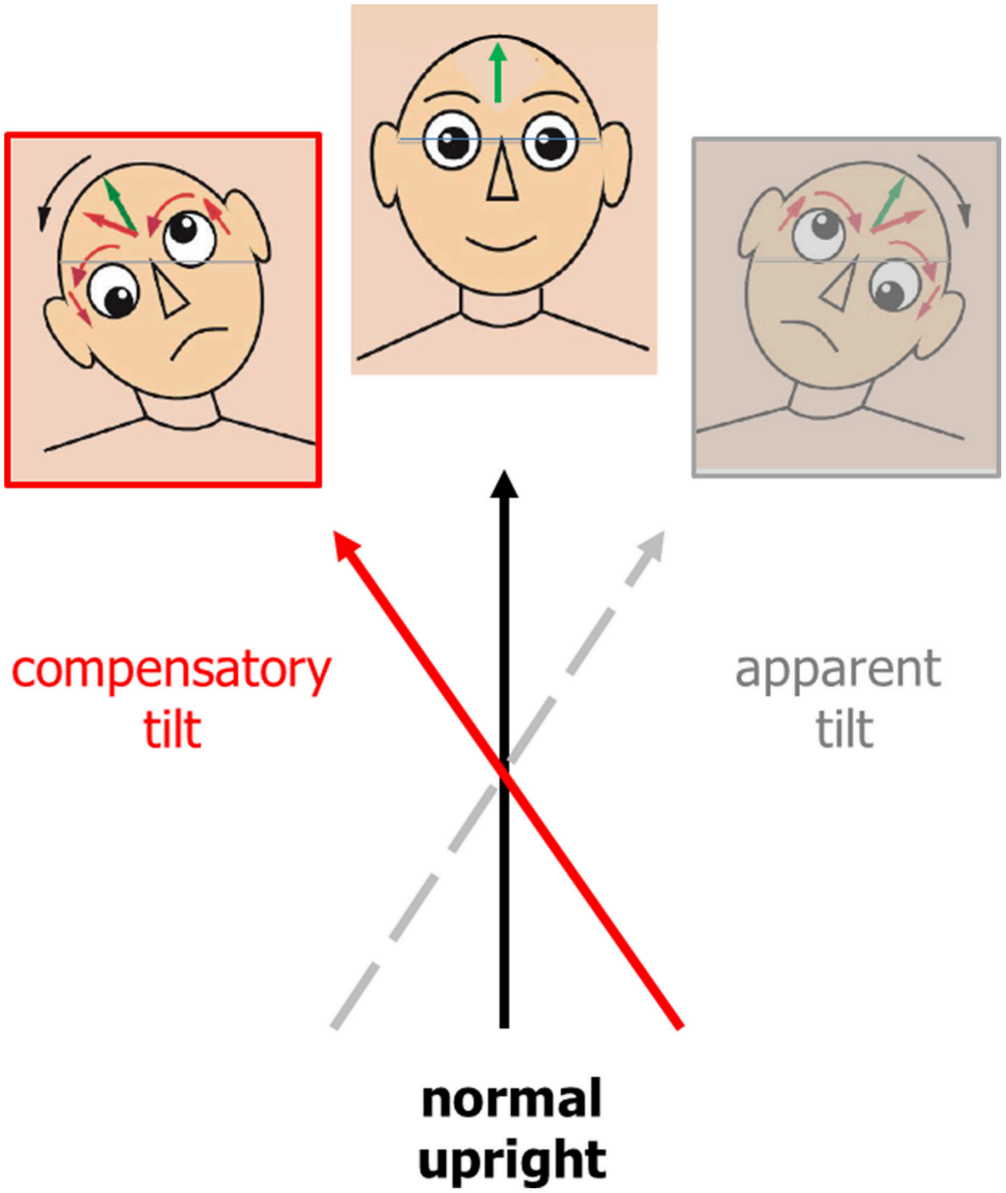
Ocular tilt reaction (i.e., triad of head tilt, vertical divergence, and ocular torsion of both eyes) and deviation of subjective visual vertical (green arrow = normal upright) represented as a “motor compensation” (in red) of a lesion-induced perception of eye-head tilt (in gray). The compensatory tilt is opposite in direction to the apparent tilt. Eyes and head are continuously adjusted to what the lesioned brain computes as being vertical.

**Figure 3:**
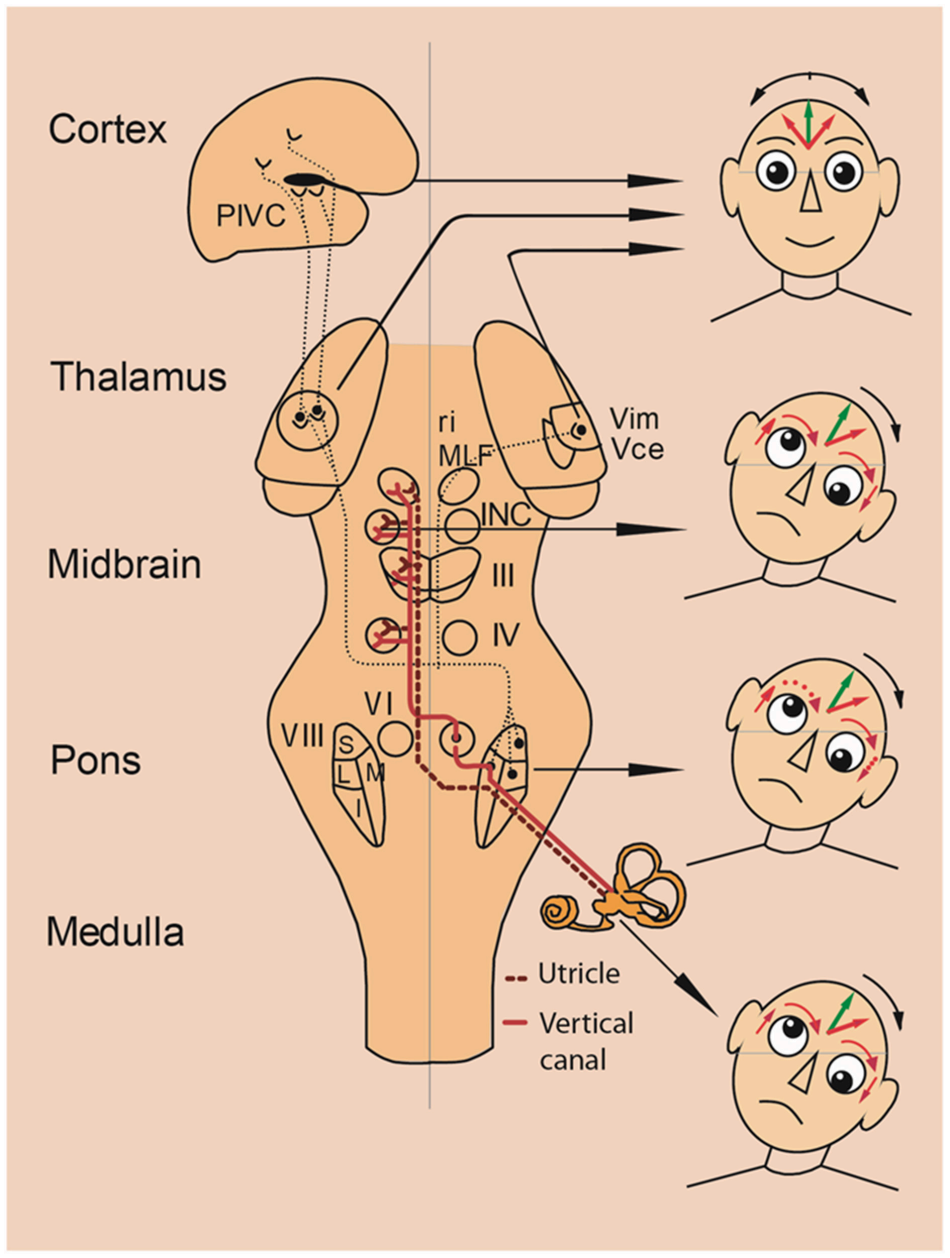
Vestibular lesions manifesting with SVV tilts and ocular tilt reaction. Pathways from the utricles and vertical semicircular canals mediate graviceptive function in the frontal roll plane. These pathways ascend from the vestibular nuclei (VIII) to the ocular motor nuclei, including the trochlear nucleus (IV), oculomotor nucleus (III) and abducens nucleus (VI). From here, they travel to the supranuclear centers of the interstitial nucleus of Cajal (INC), and the rostral interstitial nucleus of the MLF (riMLF) in the midbrain tegmentum. This circuitry is the basis for the vestibulo-ocular reflex, and is connected with vestibulospinal reflexes to control eye, head, and body posture. Projections via the thalamus (Vim, Vce) to the parieto-insular vestibular cortex (PIVC) subserve perception of verticality. Unilateral lesions of the graviceptive vestibular pathways cause vestibular tone imbalance in the roll plane. Patients with such lesions can present with an ocular tilt reaction—an eye—head synkinesis with vertical divergence of the eyes (skew deviation), ocular torsion, head tilt, and tilt of the subjective visual vertical. Right-hand images depict the resulting vestibular syndromes according to the level of the unilateral graviceptive pathway lesion. These pathways cross at the pontine level, so the direction of tilt is ipsiversive with peripheral or pontomedullary lesions (bottom two heads) and contraversive with pontomesencephalic lesions above the crossing (head at midbrain level). In thalamic and vestibular cortex lesions, there are no eye and head tilts, and tilts of the subjective visual vertical are contraversive or ipsiversive (top head). Head images: green arrows in the forehead represent objective visual vertical; red arrows represent pathological subjective visual vertical; and red arrows around the eyes represent pathological vertical deviation and torsion of the eyes. I, inferior; L, lateral; M, medial; S, superior subnuclei of the vestibular nucleus (VIII). [Modified from Brandt and Dieterich (23)].

Tilts of the visual vertical are often associated with the components of an ocular tilt reaction (OTR, an eye-head synkinesis); all tilts are in the same direction in the roll plane. The OTR consists of head tilt, skew deviation (upward deviation of one eye, downward deviation of the other), and ocular torsion combined with SVV tilts. Tilts of SVV toward the head tilt suggests that this is the perceptual correlate of perceived body tilt. The consequence is a compensatory motor response and adjustment of SVV in the opposite direction, i.e., in parallel to the direction of eye-head tilt (Figure 2). OTR was first described in monkeys (69) elicited by electrical stimulation of the unilateral mesodiencephalic structures. However, OTR can occur along the vestibular pathways from the labyrinth to the upper midbrain, but not in the thalamus and cortex (Figure 3). Due to the crossing of the graviceptive pathways in the pons the OTR is—like the SVV tilts—ipsilateral in unilateral pontomedullary lesions and contralateral in unilateral pontomesencephalic lesions, especially in those of the INC (23, 38, 52, 53).

**Figure 4:**
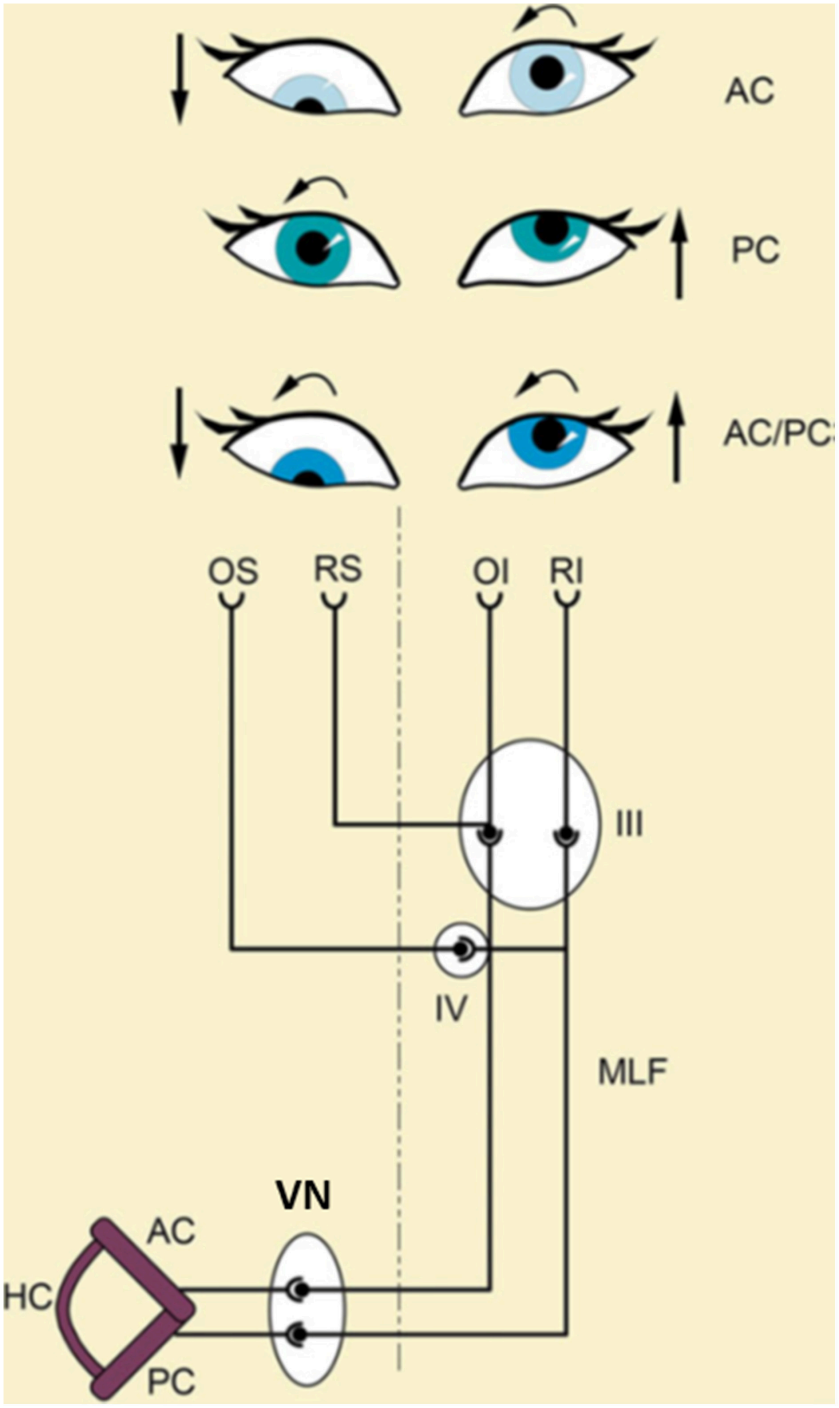
Schematical explanation of different types of ocular tilt reaction (OTR) according to the input of the affected semicircular canals. The ascending-VOR type of OTR is characterized by monocular or dysconjugate skew torsion of the eyes depending on whether input from fibers of the posterior (PC), anterior (AC), or both semicircular canals (AC/PC) to the extraocular eye muscles are affected. An excitatory ascending pathway projects from the AC to the ipsilateral superior rectus (RS) and the contralateral inferior oblique (OI) muscle. A lesion of this pathway causes a hypotropia of the ipsilateral eye and an incyclotropia of the contralateral eye (AC type; **top**). An excitatory ascending pathway is linked from the posterior semicircular canal to the ipsilateral superior oblique (OS) and the contralateral inferior rectus (RI) muscle. A lesion of this pathway causes excyclotropia of the ipsilateral eye and hypertropia of the contralateral eye (PC type; **middle**). A combination of both canals (AC/PC) induces a complete OTR (**bottom**). III, oculomotor nucleus; IV, trochlear nucleus; VN, vestibular nucleus; MLF, medial longitudinal fascicle [Modified from Brandt and Dieterich (23) and Dieterich and Brandt (35)].

Clinically there are two types of OTR (70): an “*ascending”* medullary type and a “*descending”* mesencephalic type. An OTR due to ponto-medullary vestibular nucleus lesions (Wallenberg syndrome) reflects a tone imbalance of the VOR in roll plane (Figure 4), whereas OTR caused by INC lesions (paramedian midbrain infarctions) reflects a tone imbalance of the neural integration center for vertical and rotatory eye-head coordination (54). The midbrain center not only integrates eye and head velocity for position (i.e., maintaining eye-head position in space at the end of the movement), it also adjusts vestibular reflex responses to cortical voluntary eye movements (54, 70, 71). The different manifestations of the ascending VOR type with monocular or disconjugate eye torsion indicate dysfunction of nerve fibers from the posterior, anterior, or both semicircular canals (Figure 4). If the crossed ponto-mesencephalic pathways are affected unilaterally—rostral to the downward–branching of vestibulo-spinal pathways—tilts of SVV and ocular skew-torsion occur without head tilt (23, 35). The descending mesencephalic type of OTR primarily manifests with a binocular ocular torsion. However, due to an additional damage of the trochlear or oculomotor nerve fascicles inducing monocular torsion of the ipsilateral (N. III) or contralateral eye (N. IV) the conjugate torsion can become disconjugate or monocular (30).

### SVV Tilts in Thalamic and Cortical Lesions

It is well recognized that lesions of the *thalamus*, especially of the posterolateral nuclei, induce ipsilateral or contralateral SVV tilts combined with unsteadiness of gait (54, 72). Patients with acute unilateral infarctions of these nuclei exhibit mild SVV tilts of 4–6° without any other components of OTR, i.e., without ocular torsion or skew deviation (54). Another study of the perception of verticality in 86 stroke patients reported that the thalamus is mainly involved in postural vertical perception; some of the patients manifested with pusher behavior (73). However, a specific thalamic lesion location analysis was not conducted.

To determine the distinct thalamic subnuclei associated with contralateral or ipsilateral SVV tilts, statistical lesion behavior mapping was applied in 37 stroke patients with acute circumscribed thalamic lesions (57). Two distinct regions for graviceptive processing were found: (i) Contralateral SVV tilts were caused by lesions of the nuclei dorsomedialis, intralamellaris, centrales thalami, posterior thalami, ventrooralis internus, ventrointermedii, ventrocaudales and superior parts of the nuclei parafascicularis thalami. (ii) Ipsilateral SVV tilts were caused by more inferiorly located lesions, including the nuclei endymalis thalami, inferior parts of the nuclei parafascicularis thalami, and also small parts of the junction zone of the nuclei ruber tegmenti and brachium conjunctivum (57). These data suggest separate graviceptive structures in the vestibular network which—when damaged—cause either contralateral or ipsilateral SVV tilts (57). This is in line with data from combined structural and functional connectivity mapping by means of diffusion tensor imaging combined with functional connectivity magnetic resonance imaging in right-handed volunteers (74). A link was observed between the vestibular nuclei and the ipsilateral and contralateral parieto-insular vestibular cortex (PIVC). There were five separate and distinct vestibular pathways, three of which run ipsilaterally, whereas the other two revealed a crossing at pontine or midbrain level. Of the three ipsilateral projections two run through the posterolateral or paramedian thalamic subnuclei; the third bypassed the thalamus to directly project to the inferior insular cortex (74, 75). The two contralateral pathways traveled through the posterolateral thalamus.

The disorder *thalamic astasia* is characterized by a transient postural imbalance associated with a strong tendency to fall, while motor weakness or sensory loss are absent (76). This results in lateropulsion or retropulsion. It was described in acute lesions of the posterolateral or centromedian thalamic subnuclei (54, 77, 78) and was interpreted to be a vestibular tone imbalance (54). In the few patients examined it was joined by contralateral SVV tilts (38, 78).

**Figure 5:**
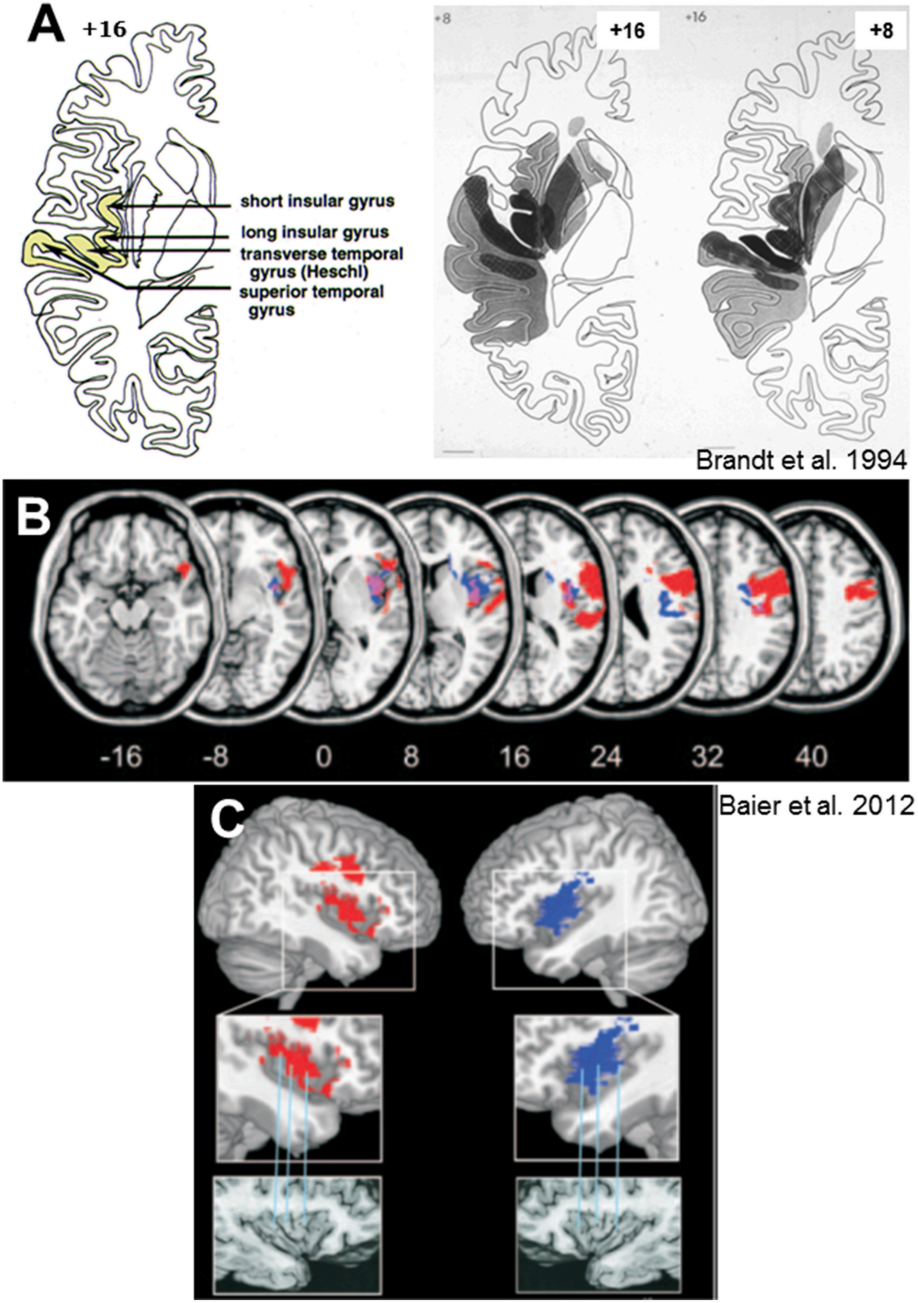
Lesion sites of hemispheric infarctions that cause tilts of subjective visual vertical. **(A)** Collective presentation of infarcted areas taken from MRI scans and projected onto sections of the atlas of Duvernoy (81) in 7 patients with clearly demarcated infarctions of the middle cerebral artery which caused significant contralateral SVV tilts. Overlapping areas of infarctions (7 of 7 in black) are centered at the posterior part of the insula, involving the short and long insular gyri, the transverse temporal gyrus, and the superior temporal gyrus [from Brandt et al. (55)]. **(B)** Statistical voxelwise lesion-behavior mapping (VLBM) analysis comparing 32 patients with acute right-sided infarctions (RBD) and 22 patients with acute left-sided infarctions (LBD) with respect to absolute tilt of subjective visual vertical (*t*-test statistic). Presented are all voxels that survived a correction for multiple comparisons using a 1% false discover rate cutoff threshold. Overlay of the statistical map from LBD patients (blue color), flipped to the right hemisphere, and the statistical map of the RBD patients (red color). Overlapping regions are shown in violet. From Baier et al. (79) **(C)** Illustration of the affected parts of the insula using the atlas of Duvernoy (81). Right insular lesions in red, left insular lesions in blue. Affected are the circular insular sulcus, central insular sulcus, short insular gyrus, and long insular gyrus [From Baier et al. (79)].

SVV deviations of about 4–6° were also seen in patients with acute *cortical* infarctions of the middle cerebral artery territory, which chiefly affected the posterior insula and the temporal gyri (55). Ocular torsion and skew deviation were not associated. With the use of voxel-based lesion behavior mapping in MRI it was possible to more precisely localize the infarction in the posterior insular cortex (e.g., long insular gyrus IV) (37, 79, 80) (Figure 5). The cortical site of the infarction causes misperception of verticality in the acute stage of stroke, thus agreeing with imaging data of patients with an acute peripheral vestibular neuritis. In the latter patients SVV tilts correlated positively with the regional cerebral glucose metabolism for the posterior insula and retroinsular region bilaterally—more so in the right hemisphere than in the left hemisphere—and for the middle temporal gyrus bilaterally (82). These studies in patients with vestibular neuritis together with the imaging data on healthy participants during galvanic vestibular (83) or visual motion stimulation, which induced circular vection (84), allowed to attribute the processing of certain aspects of the vestibular stimulus to particular parts of the vestibular thalamo-cortical network.

**Figure 6:**
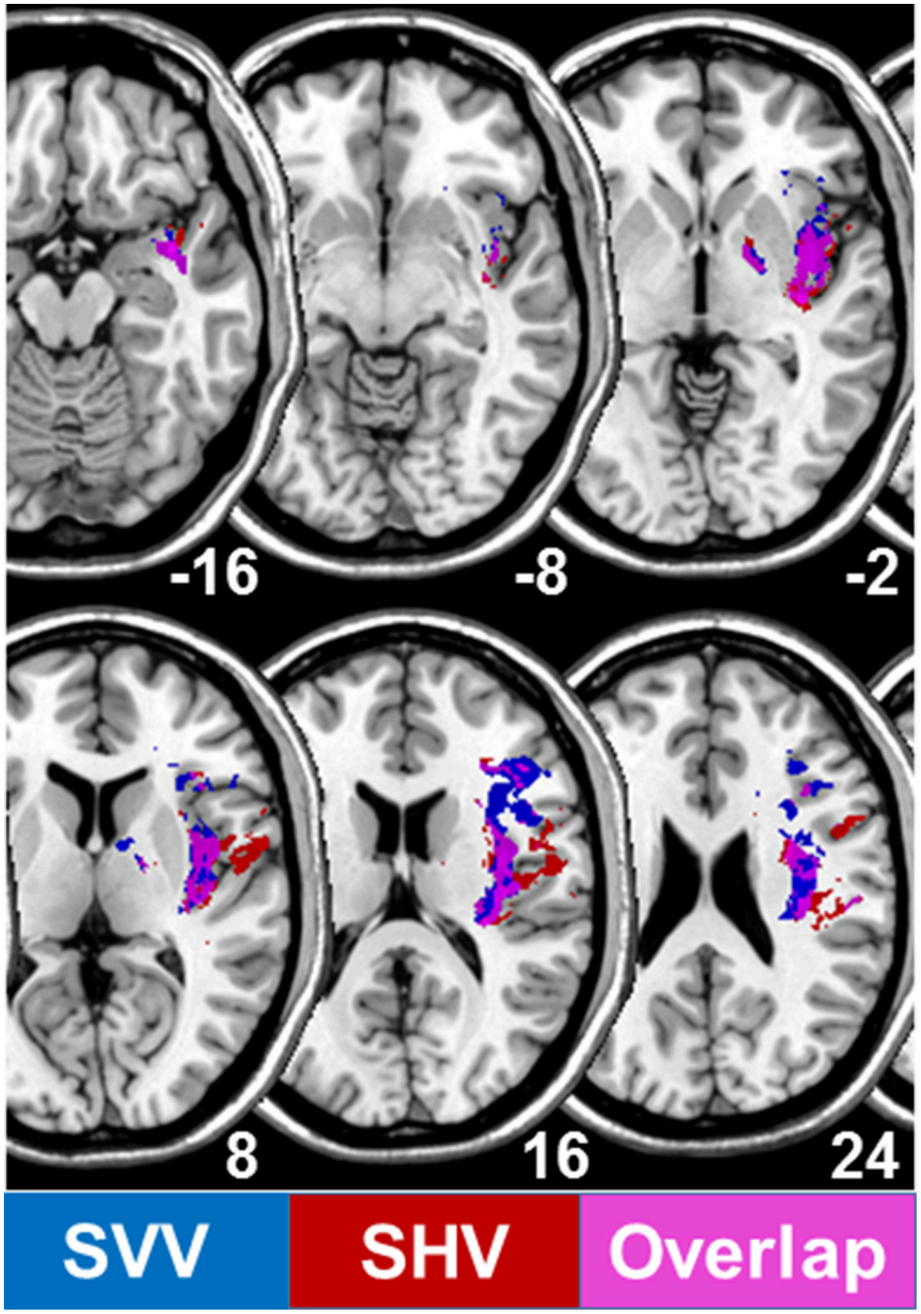
Overlapping lesion plots of 82 patients with an acute unilateral infarction of the right hemisphere. Statistical VLBM analysis comparing the 82 right brain damaged patients with respect to absolute tilts of subjective visual vertical (SVV) and subjective haptic vertical (SHV) (*t*-test statistic). Presented are all voxels that survived a correction for multiple comparisons using a 5% permutation rate correction cut off threshold. Lesions associated with SVV tilts are given in dark blue, those associated with SHV tilts in red, overlap of both in pink/violet.

**Figure 7:**
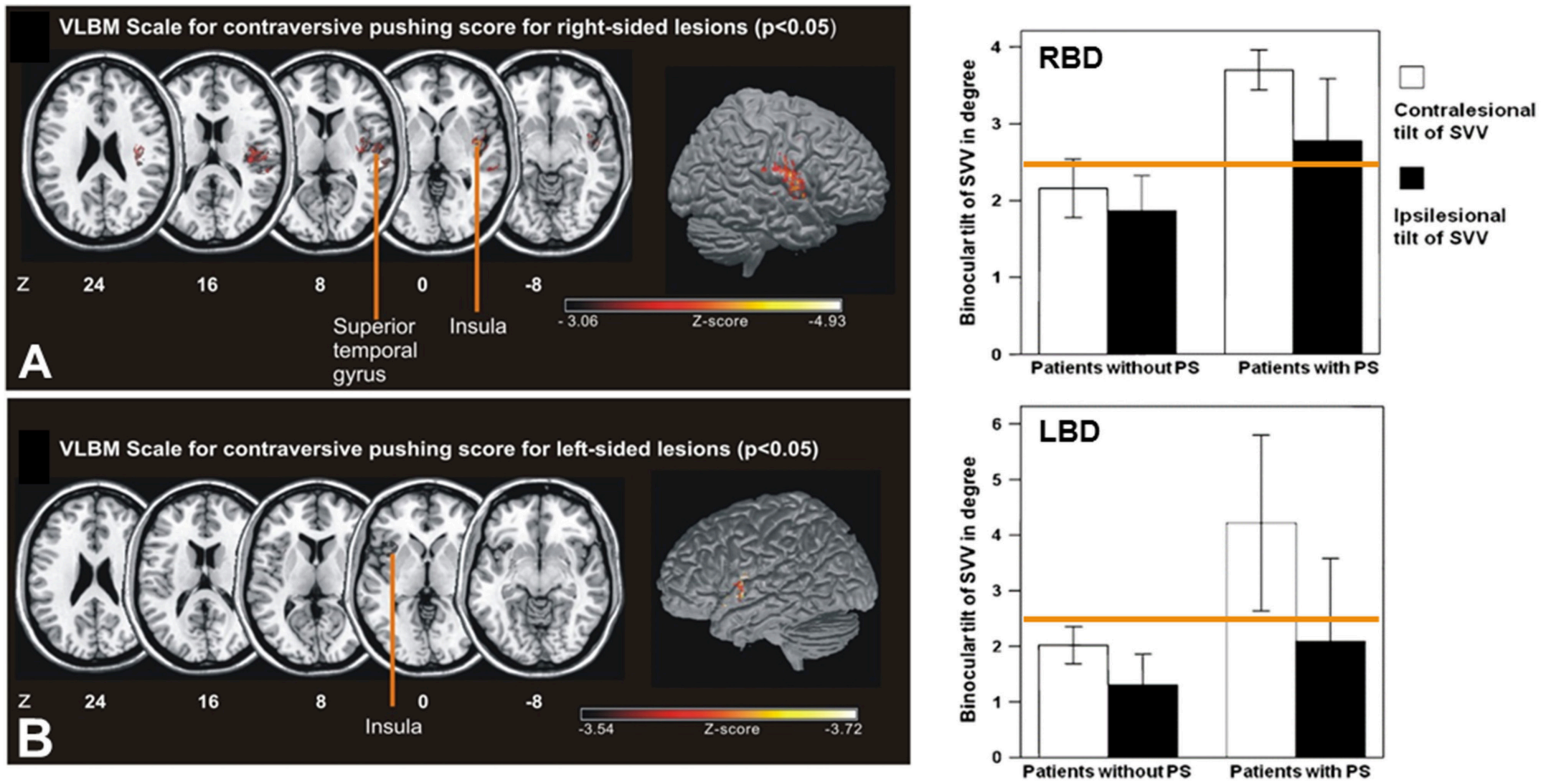
Lesion sites of acute hemispheric infarctions that cause pushing behavior. Left: Statistical voxelwise lesion-behavior mapping (VLBM) multiple regression analysis of the right-sided lesion patients (**A**: RBD; top) and left-sided lesion patients (**B**: LBD, bottom) with predictors including the Scale for Contraversive Pushing (SCP) and lesion size. The key areas of the lesion covered the posterior insular cortex, the superior temporal gyrus, and white matter in RBD. The key areas in LBD associated with the extent of contraversive pushing were the anterior insular cortex as well as parts of the operculum and the internal capsule reaching to the lateral thalamus (not shown here). Talairach z-coordinates of each transverse section are given. Right: Mean amplitude of SVV in RBD and LBD without and with pusher syndrome (PS). Significance was not obtained (*p* > 0.05) for either the contra- or the ipsilesional tilt of SVV. Error bars indicate standard error of the mean. Yellow line indicates normal range for SVV measurement [adopted in part from Baier et al. (37)].

Investigations of the SVV and the haptic vertical at later stages after right hemispheric stroke (during rehabilitation, mean day 43) showed that the lesions correlated to the SVV tilts, which occurred more centrally on the temporo-occipital junction and the posterior part of the middle temporal gyrus. The lesions correlating to the haptic tilts were located more anteriorly in the superior temporal gyrus and sulcus (21). In contrast, B. Baier from our group was able to demonstrate in patients with *acute* unilateral strokes of the right hemisphere that the lesioned areas associated with SVV tilts were found in the insular cortex, the rolandic operculum, the inferior frontal gyrus, and the frontal inferior operculum (Figure 6). Similar lesion sites were also seen in lesions associated with tilts of the haptic visual vertical located in the insular cortex, rolandic operculum, superior temporal gyrus, pallidum, Heschl's gyrus, superior longitudinal fascicle, and the corona radiate (Figure 6). An affection of insular regions and the superior temporal gyrus was also found earlier in patients with middle cerebral artery infarctions presenting with contraversive pushing (31, 37) (Figure 7).

Polysynaptic pathways and multisensory convergence link the bilaterally organized central vestibular network with cerebellar, hippocampal, limbic, and non-vestibular cortex structures to mediate “*higher” vestibular (cognitive) functions*. The cortical disorders *spatial hemineglect* and *pusher syndrome* have characteristics which can be explained by the hemispheric dominance of the vestibular network, i.e., of the right hemisphere in right-handers (85, 86).

Spatial hemineglect results from a disturbed awareness of the visual surroundings in the egocentric hemifield contralateral to an acute temporoparietal lesion (87). Stroke studies on spatial hemineglect showed that the right superior temporal cortex and the insula are preferred lesion sites (88, 89). The latter areas are parts of the distributed cortical vestibular network. Indeed, patients with spatial hemineglect exhibit systematic tilts of the SVV (90, 91). The magnitude of tilts were modulated by factors that mediate the perception of gravity and head-orientation in space (92). Neglect patients—as distinct to brain-damaged control patients—showed a counterclockwise tilt of their SVV judgments. SVV judgments were modulated by the orientation of a visible frame. If the frame was tilted counterclockwise, the spatial bias of neglect patients increased, whereas in clockwise tilts of the frame, the spatial bias decreased or even reversed in larger frame tilts (92). This enhanced rod-and-frame effect might be due to a pathologically enhanced effect of contextual visual features on SVV due to impaired processing of gravitational information (92).

Studies with vestibular caloric stimulation transiently improved spatial awareness, a finding that underlines the important contribution of cortical vestibular function in hemineglect (93, 94). When galvanic vestibular stimulation was combined with vibration of the neck muscles, the horizontal deviation of the neglect border combined linearly (95). Therefore, the spatial neglect was considered a disorder of *multisensory vestibular cortex* function (89). However, multiple sensory modalities are involved in hemineglect as well as sensorimotor control, attention, and cognition which requires multisensory integration (96). The same is true for the pusher syndrome in which somatosensory, vestibular, and visual modalities have to be integrated. Accordingly, the adjustments of SVV are influenced by the visible space and body position (97).

### Mathematical Modeling of Vestibular Function in the Roll Plane

**Figure 8:**
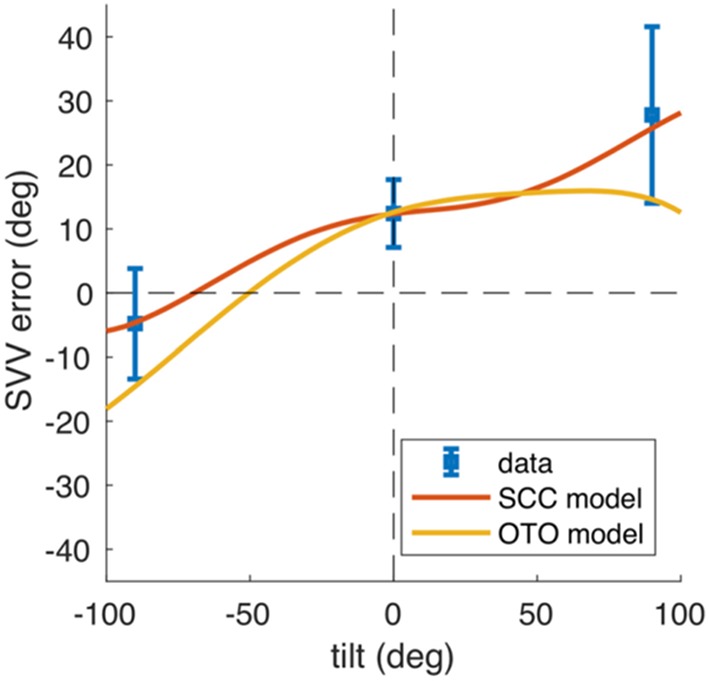
Mathematical model data predicting and simulating tilts of subjective visual vertical (SVV). SVV data from Merfeld et al. (99) obtained in patients with unilateral vestibular nerve section (blue, error bars denote SD) and model simulations of the SVV adjustments (red and yellow) in upright and tilted (right ear down, left ear down) body positions. The OTO model (yellow) assumes unequal distribution of hair cells with opposite tuning on the utricular macula (Ewald's law for otolith organs). The SCC model (red) assumes that the afferent input of vertical semicircular canals is processed centrally by the gravity estimation mechanism. After a lesion, the semicircular canal bias causes a perceptual error of gravity direction that becomes visible as SVV tilt [From Glasauer et al. (2)].

Traditionally, the effect of unilateral peripheral vestibular lesions on SVV was attributed to a tone imbalance of the otolith system (98). Use of a neural network model focusing on the direction of SVV tilts in the roll plane in upright and tilted body positions allowed comparison of the data from model simulations with clinical data (Figure 8). This recently revealed that the SVV tilt is also caused by a tone imbalance of semicircular canal input which significantly contributes to the central estimator of gravity (2). This model concept nicely confirms the earlier hypothesis that a combined dysfunction of otolith and semicircular canal input is the underlying pathomechanism of, for example, ocular tilt reaction and SVV tilt in the Wallenberg syndrome (35). The pattern of dissociated ocular torsion and skew deviation in Wallenberg patients was explained by the connections of the posterior and anterior semicircular canals with their respective plane-specific set of extraocular eye muscles (100) (Figure 4). Moreover, also the tonic ocular torsion during prolonged galvanic stimulation can be best attributed to semicircular canal activation (101). Thus, all these data allow the assumption that SVV tilts are caused by vertical semicircular canal imbalance rather than solely by an otolith imbalance. This led us to use the term “vestibular graviceptive pathways” (35).

Mathematical modeling helps understand systemic vestibular function. It requires knowledge of the neuronal circuitry, specific function of various vestibular cell systems (such as head angular velocity cells, head direction cells, or grid cells) and reliable quantitative clinical data of SVV tilts at specific lesion sites. Models should not only confirm but predict the effects of circumscribed lesions within the vestibular circuitry. Two approaches may serve as typical examples for the translational application. The first model addressed the question of why rotational vertigo is regularly caused by ponto-medullary vestibular lesions but only rarely by mesencephalic lesions (102). The second asked the question of how the different directional tilts of SVV along the ascending vestibular pathways can be explained, especially the direction-specific (ipsilateral or contralateral) tilts along the brainstem but the bilateral tilts at thalamic and cortical levels (54, 55).

The first model focused on a retrospective analysis of the frequency of rotational vertigo in acute unilateral midbrain strokes (*n* = 63) that involved the vestibular and ocular motor systems (102). Unilateral pontomedullary brainstem lesions often caused rotational vertigo, while midbrain lesions rarely caused rotational vertigo (14%) which occurred only transiently (<1 day). Swaying vertigo or unspecific dizziness (22%) and postural imbalance (31%) were typical for upper midbrain lesions. The prevailing signs were that of a vestibular tone imbalance in the roll plane in form of SVV tilts (89%), skew deviation (81%), and an incomplete or complete OTR (73%). Upper midbrain and meso-diencephalic strokes manifested chiefly with swaying or unspecific vertigo. These different manifestations were attributed to the anatomical distribution of two distinct vestibular cell systems based on semicircular canal function. The coding for head direction is performed by so-called angular head-velocity cells and head direction cells. In rodents angular head-velocity cells have been identified preferably in the lower brainstem and less frequent in the midbrain, whereas head direction cells were located mainly at midbrain and thalamic level and including cortical areas (103). The cell specific coding determines the clinical manifestation of dysfunctions of the angular velocity cell system with the sensation of body rotation and of the head direction cell system with swaying dizziness and unsteadiness. It was possible to simulate, predict, and confirm the clinical findings by mathematical modeling neural network function of the head direction cell system (102).

A subsequent model approach was used to explain the different directions of SVV tilts in the roll plane (2) (Figures 1, 3, 8). Patient studies resulted in the following topographic diagnostic rules: (i) OTR or its components are seen in unilateral lesions from the peripheral labyrinth to the midbrain including the INC. Therefore, reflexive ocular motor control by the vestibulo-ocular reflex, the head and the body by vestibulo-spinal reflexes are mediated at lower brainstem and cerebellar level (71). (ii) Lesions of the centromedial or posterolateral vestibulo-thalamic subnuclei or the parieto-insular vestibular cortex cause SVV tilts only. (iii) Lesions of thalamic or cortical vestibular areas induce both, ipsilateral or contralateral SVV tilts (54, 57, 79). These tilts are constant in a single patient, but vary interindividually (about 50% ipsilateral, 50% contralateral). (iv) It is remarkable that the degree of SVV tilts is less in thalamic and in cortical disorders as compared to peripheral or lower brainstem lesions (Figure 1, Table 1).

Other groups developed different models based on transfer functions to dynamic Bayesian inference (3–5). Here, we only refer briefly to these articles for further reading. Our review is addressed to general neurologists who are usually not educated to understand the mathematics of such an overarching conceptual model framework. The inverse probabilistic approach by Clemens and co-workers (6) is most instructive. It shows that a forward approach is difficult to implement when the different sensory inputs cannot be studied in isolation. Their model predictions are based on the derived noise properties from the various modalities (6). They found that the accuracy of orientation estimates of subjective body and visual vertical in healthy subjects can be linked to a reference-frame-dependent weighting of sensory signals (6). This reverse-engineering approach in the healthy subjects was consistent with published data of two patients groups with acquired neurological or vestibular disorders (10, 11), which led them to speculate on the clinical relevance of such models. Furthermore, recent experiments emphasize the role of vestibular cerebellar function for gravity perception (104).

Why do SVV tilts at thalamic and cortical levels differ from those at brainstem level? One explanation could be that a partial crossing of the ascending pathways in the midbrain at the level of the INC provides the thalamus and the cortex with graviceptive input from both labyrinths (74). This enables the bilateral thalamocortical networks to operate separately in the right and left hemisphere because there is no direct interconnection between the two thalamic nuclei complexes (14, 105). An alternative or supplementary explanation could be based on different neuronal coding principles for graviceptive input due to the different vestibular cell systems, according to the discussed head direction cell system for the horizontal yaw plane (103). Findings in the macaque monkey concerning the tuning of gravity in anterior thalamic neurons (106) confirm this view. An analysis of 15 studies (2) on the effects of unilateral peripheral or central vestibular lesions on the direction and amount of SVV tilts showed the following findings (see Table 1): acute unilateral labyrinthine or eighth nerve lesions caused ipsilateral SVV tilts in upright head and body position. Maximal tilts were found in complete vestibular loss caused by labyrinthectomy or neurectomy (Figure 1).

The gravity coding which changes from a peripheral or brainstem vectorial representation in otolith coordinates to a coding of distributed population at thalamic and cortical levels is compatible with the affects of unilateral thalamic and cortical lesions that variably effect the perceived verticality. This population-coding network for the perception of the gravity vector implements the elements that are required for the described perceptual underestimation of the SVV in tilted body positions, i.e., the Aubert effect (2) (Figure 8).

## Conclusion

Thus, it is the level of the lesion of graviceptive vestibular pathways which is critical for the control of verticality perception and the position of eye, head, and body relative to gravity in the roll plane. It explains all features of OTR including postural instability:

- Medullary lesions may cause lateropulsion.
- Vestibular nucleus lesions cause ipsilateral “VOR-OTR” with monocular or disconjugate ocular torsion.
- Brainstem lesions between the vestibular nucleus and the rostral midbrain cause SVV tilts and ocular skew-torsion.
- INC lesions cause “integrator OTR” with binocular conjugate ocular torsion.
- Unilateral vestibular lesions above brainstem level from meso-dienecephalic vestibular structures to the cortex as a rule manifest with perceptual rather than motor dysfunctions.
- Lesions at thalamic level cause SVV tilts without associated ocular motor signs; rarely vestibular thalamic astasia may occur.
- Lesions of the insular and temporo-parietal cortex cause mild ipsilateral or contralateral SVV tilts and in exceptional cases transient vertigo.

Patients with cortical lesions of the vestibular system may also present with higher vestibular dysfunctions such as visuospatial hemineglect and pusher syndrome. Higher vestibular dysfunctions involve cognition and more than one sensory modality. They involve multisensory convergence and sensorimotor interaction for spatial memory, spatial orientation, navigation, and attention. Based on the clinical data, mathematical models have been developed which are able to simulate and predict the deficits of gravity perception in patients with neurological and otoneurological disorders.

## Author Contributions

All authors listed have made a substantial, direct and intellectual contribution to the work, and approved it for publication.

### Conflict of Interest Statement

The authors declare that the research was conducted in the absence of any commercial or financial relationships that could be construed as a potential conflict of interest.

## Abbreviations

SPV: subjective postural vertical
SVV: subjective visual vertical
OTR: ocular tilt reaction
INC: interstitial nucleus of Cajal
PSP: progressive supranuclear palsy
NPH: normal pressure hydrocephalus
riMLF: rostral interstitial nucleus of the medial longitudinal fascicle.
